# Vitamin D alleviates obesity-related metabolic abnormalities by modulating the gut microbiota in older female mice on a high-fat diet

**DOI:** 10.3389/fcimb.2025.1703497

**Published:** 2025-12-10

**Authors:** Dandan Li, Dongmei Liu, Yali Wang, Qian Xu, Tao Wang, Pengsha Sun, Xinjun Yu

**Affiliations:** 1 Department of Geriatrics, Affiliated Hospital of Shandong Second Medical University, Weifang, China; 2 Clinical Research Center, Affiliated Hospital of Shandong Second Medical University, Weifang, China; 3 School of Clinical Medicine, Affiliated Hospital of Shandong Second Medical University, Weifang, China

**Keywords:** vitamin D, older, lipid metabolism, gut microbiota, intestinal homeostasis

## Abstract

**Introduction:**

Older women experience a significant decline in estrogen levels due to ovarian dysfunction, leading to a series of health issues such as lipid metabolism disorders, obesity, and increased risk of cardiovascular disease. Previous studies have shown that vitamin D deficiency can increase the risk of metabolic diseases.

**Methods:**

This study used older female mice fed a high-fat diet as research subjects to investigate the effects of vitamin D on lipid metabolism abnormalities in older female mice and whether these effects are related to the regulation of the gut microbiota.

**Results:**

Our results indicate that vitamin D supplementation reduces body weight, blood lipid levels, and mild inflammation in older female mice, improves hepatic steatosis and fibrosis, regulates the expression of fatty acid metabolism genes, and increases the expression of tight junction proteins in the gut. HepG2 fatty liver cells also validated these findings. Gut microbiota sequencing results showed that vitamin D supplementation significantly regulated the overall composition of the gut microbiota, reducing the abundance of microbiota associated with obesity and inflammation, increasing the abundance of beneficial bacteria, and reversing gut microbiota dysbiosis caused by a high-fat diet. Additionally, Spearman correlation analysis indicated that key microbial communities regulated by vitamin D were highly correlated with metabolic markers.

**Conclusions:**

These results suggest that vitamin D could serve as a potential candidate drug for preventing lipid metabolism abnormalities caused by obesity in older female mice by regulating the gut microbiota.

## 1 Introduction

Older women experience a decline in estrogen levels due to ovarian dysfunction, which increases the risk of obesity, metabolic syndrome, and non-alcoholic fatty liver disease (NAFLD) (Duralde et al., 2023). The decrease in estrogen further promotes inflammatory responses, with elevated levels of pro-inflammatory cytokines (IL-1, TNF-α) in the serum of postmenopausal women (Vrieling and Stienstra, 2023). Studies have shown that the increased prevalence of NAFLD is associated with aging, with a prevalence rate as high as 57.9% in older women (Razmjou et al., 2018).

Vitamin D deficiency is associated with the development of cardiovascular disease, metabolic syndrome, and hyperlipidemia (Karampela et al., 2021; Li et al., 2024). Ma et al. found that serum 25(OH)D was negatively correlated with carotid atherosclerosis in postmenopausal women with normal blood pressure and glucose tolerance (Ma et al., 2014). A cohort study found that vitamin D deficiency in older women was associated with a high prevalence of metabolic syndrome (Schmitt et al., 2018). Vitamin D reduces cytokine release and adipose tissue inflammation by inhibiting NF-κB signaling (Guan et al., 2022). Most of the functions of vitamin D are mediated by vitamin D receptor (VDR), which are widely distributed in tissues and cells in the body (Wang et al., 2024). It has been found that serum cholesterol increases in both male and female mice when VDR is knocked out (Abbas, 2017).

The gut microbiota is a complex and dynamic ecosystem that regulates the permeability of the gastrointestinal mucosa and the host immune system (Martinez and Grant, 2025). It is well known that the gut microbiota is associated with obesity and metabolic syndrome, with obese individuals having a gut microbiota composed of fewer Bacteroidetes and more Firmicutes (Rizzoli et al., 2021; Thibaut and Bindels, 2022; Zhang et al., 2025). Probiotics containing *Bifidobacterium* and *Lactobacillus* can help reduce body weight in mice fed a high-fat diet and improve lipid and glucose homeostasis (Thomas et al., 2020; Kuziel and Rakoff-Nahoum, 2022). Additionally, an imbalance in the composition of the gut microbiota may impair intestinal barrier function, increase endotoxin levels in the circulatory system, and ultimately lead to obesity (Bradley and Haran, 2024). Vitamin D is a key regulator of intestinal physiology and internal environmental homeostasis, playing a crucial role in maintaining intestinal homeostasis (Armet et al., 2022).

To our knowledge, there are currently no published studies on the effects of vitamin D on metabolic abnormalities in older female mice, nor on whether such results are related to the gut microbiota. In this study, we found that vitamin D ameliorates obesity and metabolic abnormalities in high-fat diet-induced elderly female mice by modulating the gut microbiota, increasing the relative abundance of beneficial bacteria, reducing inflammation, and maintaining the intestinal epithelial barrier.

## 2 Materials and methods

### 2.1 Animal experiment design

Twenty-four female C57BL/6 mice (aged 18–20 months, weighing over 20 g) were purchased from Vital River Laboratory Animal Technology (Beijing, China) (Chen et al., 2025). The mice were housed in a 25 °C specific pathogen free (SPF) room with free access to water and food. Mice in the diestrus phase were selected for the experiment based on vaginal cytology smears. Twenty-four older female mice were randomly divided into three groups (eight mice per group): (1) normal diet group (NC), (2) high-fat diet group (HFD), and (3) high-fat diet+vitamin D group (VD): 1,25(OH)_2_D_3_ (Sigma-Aldrich Co., St. Louis, MO, USA) was dissolved in corn oil, with an oral dose of 1000 U/kg/day per mouse (Muñoz-Aguirre et al., 2015), and maintained for 12 weeks. Food intake and body weight were recorded daily. After the experiment, vaginal cytology smears were performed on mice. Additionally, fecal samples were collected from mice in each group under sterile conditions for intestinal microbiota sequencing analysis. The mice were fasted for 12 hours, anesthetized with sodium pentobarbital (150 mg/kg body weight), followed by cervical dislocation for euthanasia, and blood was collected from the posterior orbital plexus. Liver, fat and colon tissues were weighed and stored at -80 °C for further analysis. The components of the high-fat diet and standard diet are shown in **Supplementary Table S1**. We adhered to ethical guidelines for the treatment of laboratory animals, and this study was approved by the Institutional Animal Care and Use Committee (IACUC).

### 2.2 Biochemical analysis and intraperitoneal glucose tolerance test

Serum triglycerides (TG), total cholesterol (TC), low-density lipoprotein cholesterol (LDL-c), high-density lipoprotein cholesterol (HDL-c), alanine transaminase (ALT), and aspartate transaminase (AST) in the serum of each group of older female mice were measured using a commercial assay kit (Elabscience Biotechnology Co., Ltd., Wuhan, China) in strict accordance with the manufacturer instructions. IPGTT experiments were next performed on groups of mice. After overnight fasting, mice in each group were injected intraperitoneally with glucose solution, and blood was collected from the tail vein and measured for blood glucose concentration at different time intervals.

### 2.3 Enzyme linked immunosorbent assay

Serum levels of follicle-stimulating hormone (FSH), luteinizing hormone (LH), and 25(OH)D were measured using an ELISA assay. The ELISA kit was purchased from Elabscience (Wuhan, China) and used according to the manufacturer instructions. Further ELISA kits were used to detect cytokine levels (IL-1β, IL-6, and TNF-α) in serum and liver tissue, with OD values measured at 450 nm using an enzyme-linked immunosorbent assay reader (Enspire, Perkin-Elmer, Singapore).

### 2.4 Histological analysis

Fresh liver and adipose tissue were dissected and rinsed with physiological saline to remove blood stains. The tissue was then immersed in 4% paraformaldehyde for 48 h for histological examination. The tissue samples were sectioned, dewaxed, stained with hematoxylin and eosin (H&E), and sealed with neutral resin. Finally, under a microscope (Nikon Eclipse BO, Japan) at 200× magnification, histological changes in liver and fat tissue were analyzed using ImageJ software (NIH, Bethesda, MD, USA).

### 2.5 RNA isolation and real-time PCR

Total RNA was extracted from liver and colon tissues using TRIzol reagent (Invitrogen, Carlsbad, CA, USA). RNA concentration was determined using a Nanodrop 2000c spectrophotometer (Thermo Fisher Scientific, Waltham, MA, USA). Reverse transcription was performed using the PrimeScript RT Reagent Kit (Takara, Otsu, Japan) according to the manufacturer instructions to convert total RNA into complementary DNA (cDNA). RT-PCR analysis was performed using the SYBR Premix Ex Taq Kit (Takara, Otsu, Japan). Samples were analyzed in triplicate, with β-actin used for mRNA expression normalization, and relative gene expression levels were determined using the 2^-ΔΔCt^ method. Primer designs are shown in **Supplementary Table S2**.

### 2.6 Special staining

After deparaffinization and hyalinization of the liver tissue, the tissue sections were stained with Weigert iron hematoxylin for 8 min, followed by differentiation with hydrochloric acid alcohol. Further staining was performed with Masson trichrome stain (Solarbio, China) for 5 min. The sections were then washed with ice-cold acetic acid solution, dehydrated, and cleared, followed by neutral balsam mounting. The sections were observed under a microscope and photographed for documentation. Next, the vaginal secretions of each group of mice were stained with Wright-Giemsa staining solution (Baso, China). Solution A and B were added to the smear dropwise and stained for 10 min. Finally, it was rinsed, dried and observed under the microscope.

### 2.7 Cell experiments

Human hepatocellular carcinoma (HepG2) cells were purchased from the American Type Culture Collection (ATCC). HepG2 cells were cultured in DMEM medium (Gibco, America) containing 10% fetal bovine serum (Gibco, America) and 1% penicillin/streptomycin (Procell, China) and incubated in a 37 °C, 5% CO2 incubator (Thermo Fisher Scientific, USA). The optimal experimental concentration of 1,25(OH)_2_D_3_ was determined using the Cell Counting Kit-8 (CCK8) (Yeasen Biotechnology Co., Ltd., Shanghai, China). HepG2 cells were treated with different concentrations of 1,25(OH)_2_D_3_ (10-^10^-10-^6^ M). HepG2 cells were further seeded into 6-well plates and cultured in DMEM medium containing 5% fetal bovine serum for 24 h. The cells were then divided into control (CON), high fat (HF, palmitic acid: oleic acid = 1:2) and high fat+vitamin D (VD) groups. Finally, the cells were stained with Oil Red O solution (Solarbio, China) and the cell morphology was observed under the microscope. Simultaneously, RNA of each group was extracted to detect the mRNA level of target genes.

### 2.8 Western blot

An appropriate amount of colon tissue was rinsed and weighed, and RIPA lysis buffer (Solarbio, China) was added to grind on ice to extract the total protein. The concentration of the protein sample was determined by the BCA protein assay kit (Solarbio, China). Electrophoresis was performed using SDS-PAGE gels at a concentration of 10%. Then, the gel is placed on a PVDF membrane (Immobilon^®^-P, Merck Millipore, Darmstadt, Germany) activated with formaldehyde. After 5% skim milk blocking, incubation with primary and secondary antibodies, and finally visualization of the washed PVDF membranes using the ChemiDoc™XRS system from Bio-Rad (Shanghai, China). The gray bands were quantified by Image J software. The primary antibody dilutions used in this study were: claudin-1 (1:1000), occludin (1:1000), β-actin (1:1000).

### 2.9 Gut microbiome analysis

Under aseptic environment, feces of each group of mice were collected and stored at -80 °C for further analysis of the gut microbiota. Fecal DNA was extracted using the fecal DNA extraction kit (TIANGE, Beijing, China) and then detected by 1% agarose gel electrophoresis. Mouse fecal 16S rRNA sequencing was performed at Origingene (Origingene Bio-pharm Technology Co., Ltd., Shanghai, China). The sequencing platform utilized was Illumina HiSeq, with paired-end reads of 250 bp. The sequencing region targeted the 16S rRNA V3-V4 zone, achieving an average sequencing depth of 40,000 reads per sample. All samples attained a sequencing coverage rate of≥97%. Analysis and plotting were performed using R software (Version 2.15.3). The Chao1 index was used to calculate microbial abundance, and the Shannon index was used to calculate microbial diversity. PCoA analysis was used to distinguish differences in microbial composition between samples. Spearman correlation analysis was used to study the correlation of gut microbiota with metabolic parameters, intestinal mucosal barriers, and other biological functions at the genus-species level.

### 2.10 Statistical analysis

Data analysis was performed using SPSS 19.0 statistical software. All data were presented as mean ± standard deviation (mean ± SD) of at least three independent experiments, and Student’s T-test was used for data analysis between two groups, and one-way ANOVAs analysis of variance was used for data analysis between multiple groups. *P*<0.05 was considered statistically significant.

## 3 Results

### 3.1 Effects of vitamin D on weight, blood sugar, and hormones

**Figure 1:**
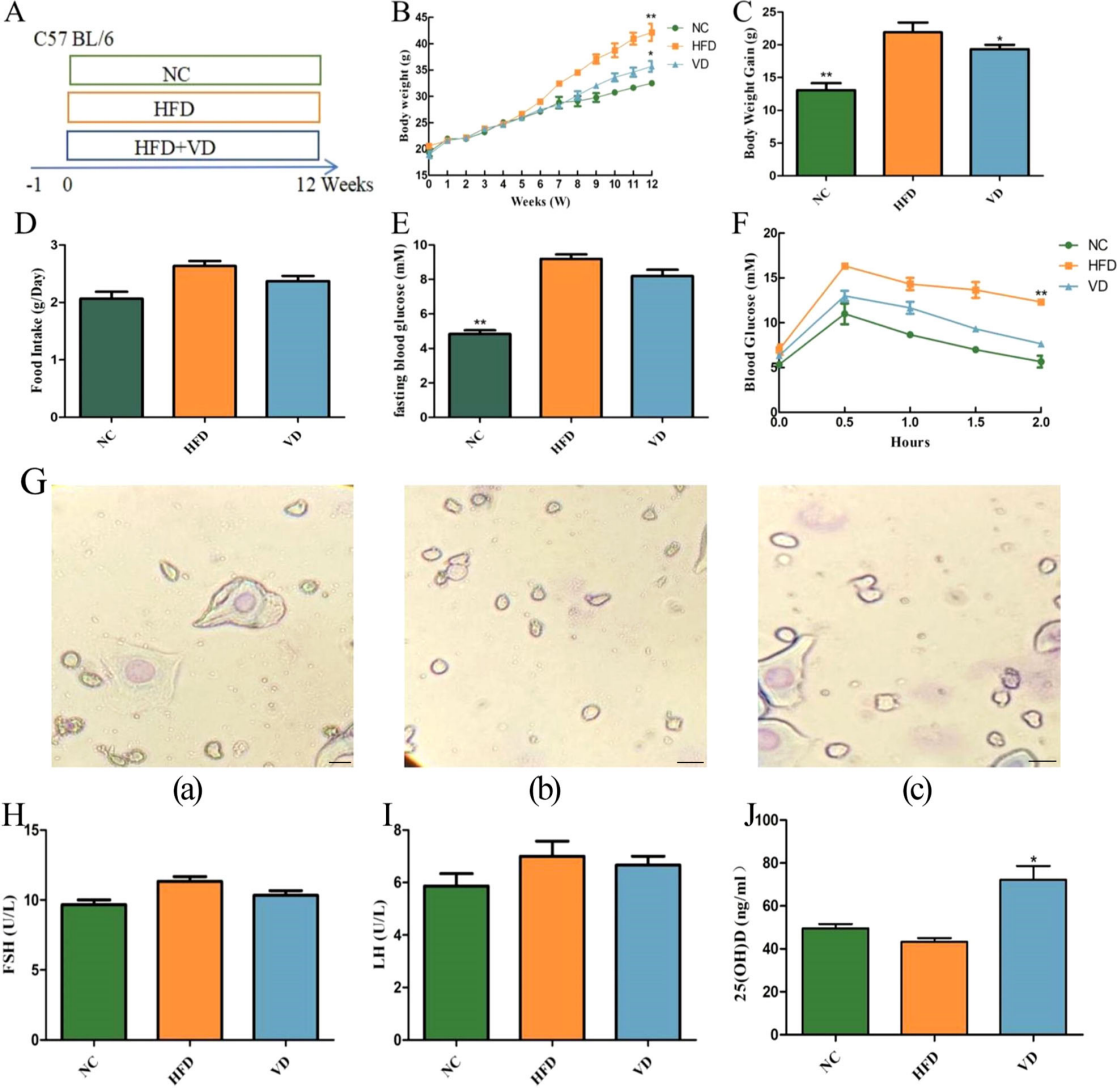
The effects of vitamin D on body weight, blood glucose, and hormones. **(A)** Experimental design diagram. **(B)** Weight changes. **(C)** Weight gain. **(D)** Food intake. **(E)** Fasting blood glucose. **(F)** IPGTT. **(G)** Giemsa staining (100×): NC group (a), HFD group (b), VD group (c), Plotting scale=100 μm. **(H)** Serum FSH. **(I)** Serum LH. **(J)** Serum 25(OH)D. Data are the mean ± SD (n=8). Compared with the HFD, *P<0.05 and **P<0.01.

Older female mice were screened out by vaginal cytology smears for experiments, and the experimental flow is shown in **Figure 1A**. Starting from week 6, mice in the HFD group showed a trend toward weight gain (**Figure 1B**). After 12 weeks, the weight of mice in the HFD group increased significantly compared with that of mice in the NC group (*P*<0.01), with an increase of approximately 33% (**Figure 1C**). Compared with the HFD group, the weight of mice in the VD group decreased (*P*<0.05). In terms of food intake, there was no statistically significant difference between the NC and VD groups compared to the HFD group (**Figure 1D**). We further assessed the effect of vitamin D supplementation on glucose homeostasis. As shown in **Figures 1E, F**, fasting blood glucose levels in the HFD group were significantly higher than those in the NC group (*P*<0.01), but VD group did not improve HFD-induced hyperglycemia. Giemsa staining showed that the vaginal cell smears of all three groups of mice showed a large number of small, round leucocytes and a few nucleated epithelial cells, indicating that they were in the diestrus phase (**Figure 1G**). Serum FSH and LH levels were elevated in all groups of mice, and the differences were not statistically significant (**Figures 1H, I**). Finally, serum 25(OH)D levels were compared among the groups. As shown in **Figure 1J**, compared with the HFD group, serum 25(OH)D levels increased in the VD group after 12 weeks of vitamin D administration (*P*<0.05), while there were no significant changes in serum 25(OH)D levels in the NC and HFD groups.

### 3.2 Effects of vitamin D on adipose tissue and blood lipids

**Figure 2:**
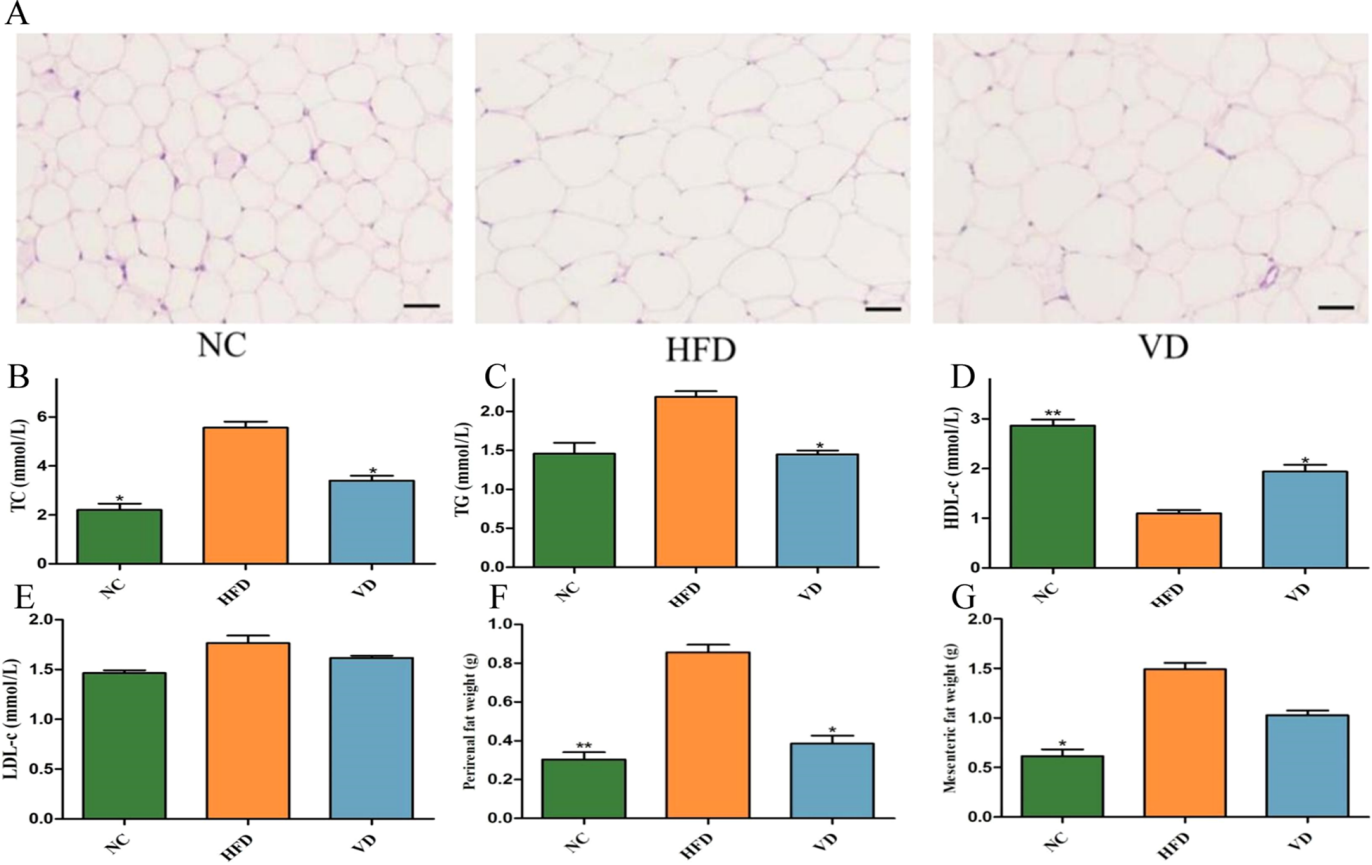
Effect of vitamin D on adipose tissue and blood lipids. **(A)** Adipose tissue HE staining, Plotting scale=100 μm. **(B)** Serum TC. **(C)** Serum TG. **(D)** Serum HDL-c. **(E)** Serum LDL-c. **(F)** Perirenal fat weight. **(G)** Mesenteric fat weight. Data are the mean ± SD (n=8). Compared with the HFD, *P<0.05 and **P<0.01.

As shown by HE staining of adipose tissue (**Figure 2A**), the adipocytes of mice in the HFD group were larger than those in the NC group, but the adipocyte volume decreased after vitamin D supplementation. Next we tested the lipid levels in each group of mice. As shown in **Figures 2B–E**, compared with the NC group, serum TC levels were elevated in the HFD group (*P*<0.05), TG levels showed a trend toward elevation but were not statistically significant, and HDL-c levels were significantly reduced (*P*<0.01), decreasing by approximately 65%. Compared with the HFD group, vitamin D supplementation reduced serum TC levels (*P*<0.05) and TG levels (*P*<0.05), increased serum HDL-c levels (*P*<0.05), and showed a trend toward reducing LDL-c levels, but the difference was not statistically significant. We further analyzed the weights of mesenteric fat and perirenal fat in each group of mice and found that the weights of mesenteric fat and perirenal fat in the HFD group were significantly higher than those in the NC group (*P*<0.05). Vitamin D supplementation significantly reduced perirenal fat weight (*P*<0.05) and showed a trend toward reducing mesenteric fat weight, but the difference was not statistically significant (**Figures 2F, G**).

### 3.3 The effect of vitamin D on cytokine and fatty acid metabolism genes

**Figure 3:**
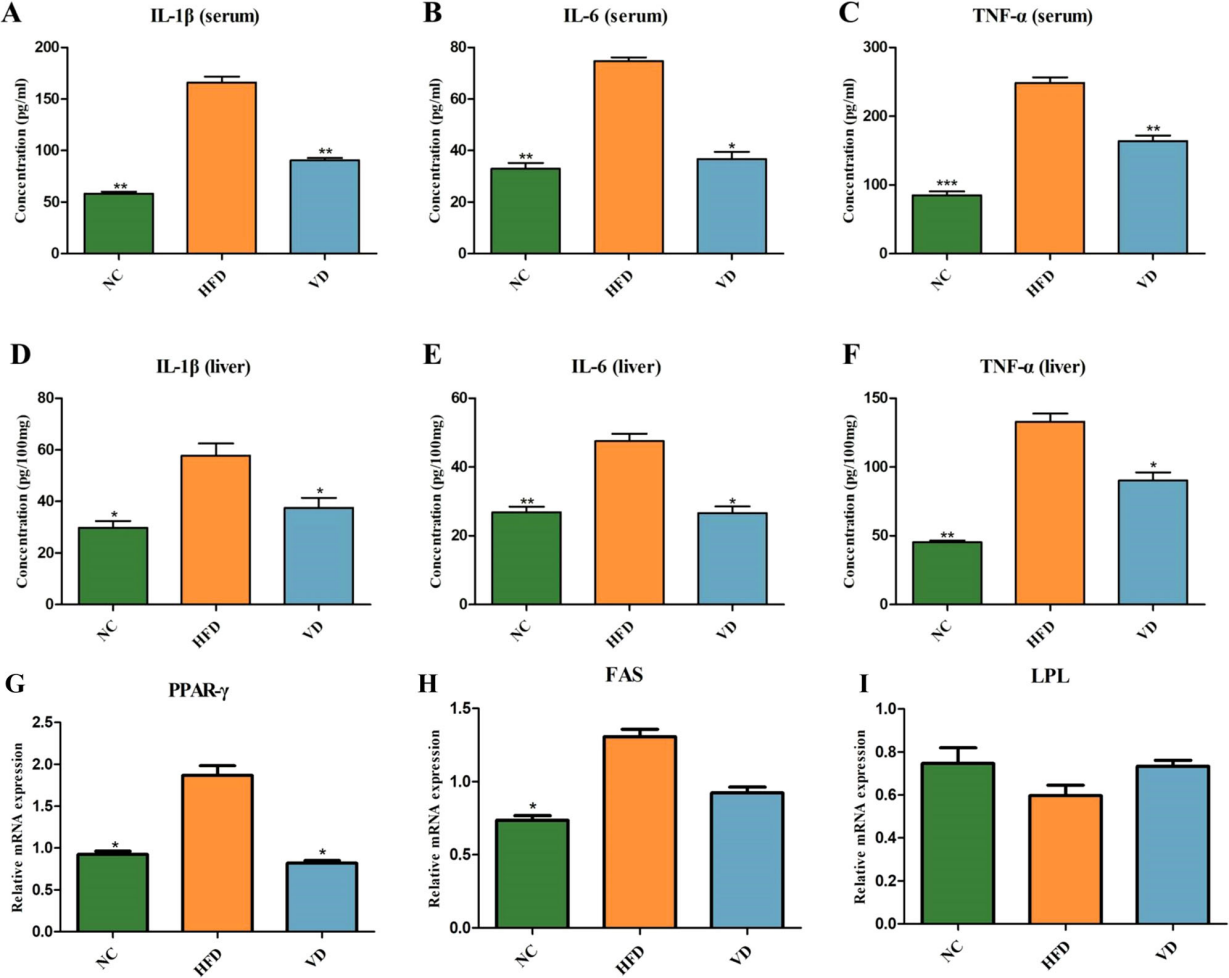
Serum levels of cytokines IL-1β **(A)**, IL-6 **(B)**, and TNF-α **(C)**. Liver tissue levels of cytokines IL-1β **(D)**, IL-6 **(E)**, and TNF-α **(F)**. Liver tissue expression of *PPAR γ* mRNA **(G)**, *FAS* mRNA **(H)**, and *LPL* mRNA **(I)**. Data are the mean ± SD (n=8). Compared with the HFD, *P<0.05, **P<0.01 and *** *P*<0.001.

The levels of cytokines in serum and liver tissues are shown in **Figures 3A–C**. Serum levels of IL-6, IL-1β and TNF-α were significantly higher in the HFD group compared to the NC group (*P*<0.01). Cytokine levels in liver tissue were similar to those in serum (**Figures 3D–F**). As expected, vitamin D supplementation reduced serum IL-6 levels (*P*<0.05). Notably, vitamin D had a more pronounced effect on reducing serum IL-1β and TNF-α levels (*P*<0.01). In liver tissue, vitamin D supplementation similarly reduced pro-inflammatory cytokine levels (IL-6, IL-1β, and TNF-α) compared to the HFD group.

To further understand the molecular mechanism by which vitamin D protects the liver, we measured the expression levels of fatty acid metabolism genes peroxisome proliferator-activated receptor γ (PPAR γ), fatty acid synthase (FAS), and lipoprotein lipase (LPL) in liver tissues. As shown in **Figures 3G–I**, the expressions of *PPAR γ* mRNA (*P*<0.05) and *FAS* mRNA (*P*<0.05) in liver tissues in the HFD group were increased compared with the NC group. Vitamin D could reduce the expression of *PPAR γ* mRNA in liver tissue (*P*<0.05), but vitamin D had no significant effect on the expression of *LPL* and *FAS* mRNA.

### 3.4 The effect of vitamin D on liver tissue and HepG2 fatty liver cell models

**Figure 4:**
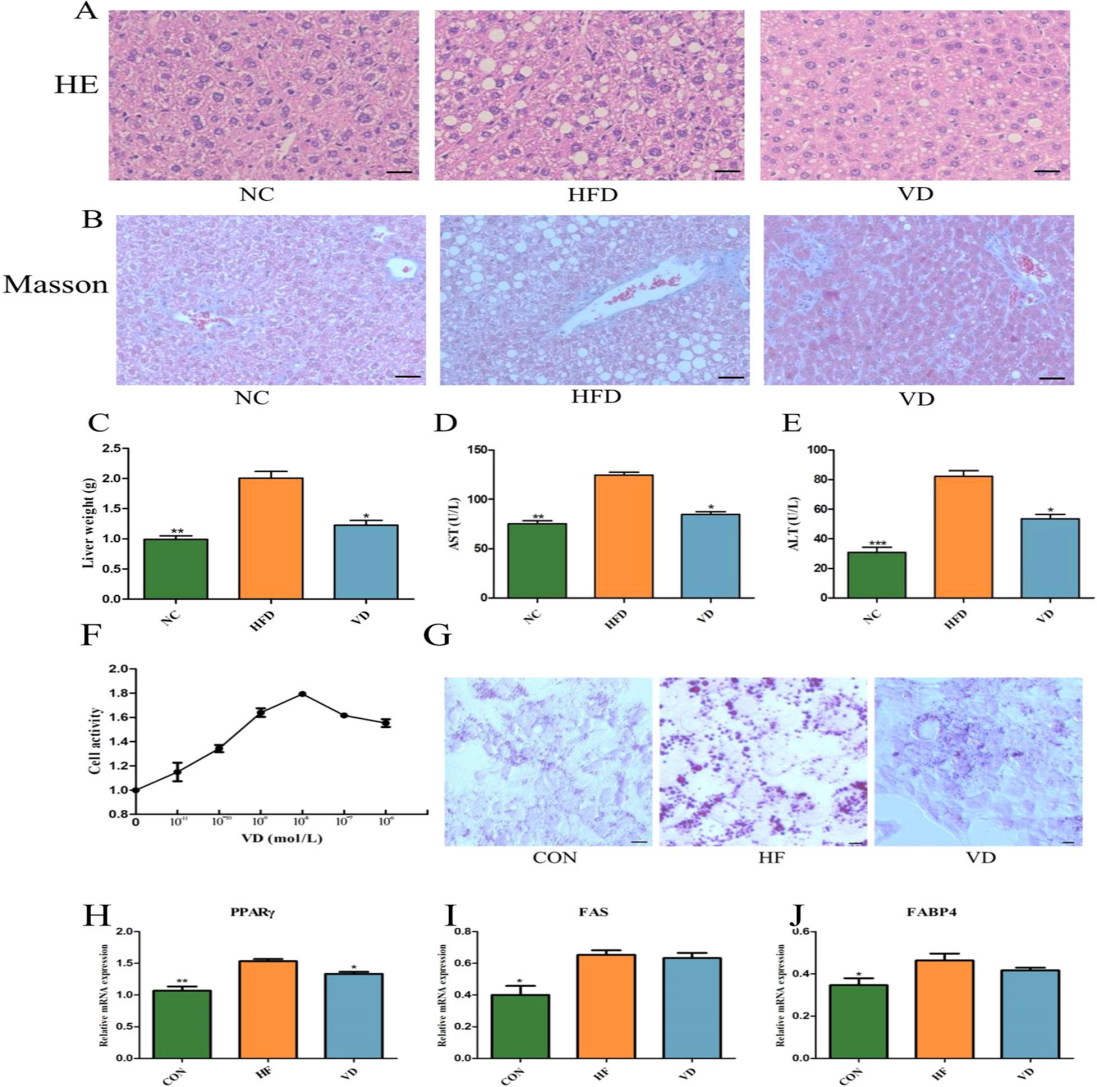
Liver tissue pathology and HepG2 fatty liver cell model. **(A)** Liver HE staining. **(B)** Liver Masson staining. **(C)** Liver weight. **(D)** Serum AST. **(E)** Serum ALT. **(F)** CCK-8. **(G)** Oil Red O staining. RT-PCR analysis of *PPAR γ***(H)**, *FAS***(I)**, and *FABP 4***(J)** mRNA expression in HepG2 cells. Plotting scale=100 μm. Data are the mean ± SD (n=8). Compared with the HFD, *P<0.05, **P<0.01 and *** *P*<0.001.

The results of HE staining of liver tissues in each group shown in **Figure 4A**. No obvious fat vacuoles were observed in the livers of the NC group. In the HFD group, hepatocytes were swollen, with fat vacuoles of varying sizes appearing in the cytoplasm, and the arrangement of hepatic trabeculae was disordered, with inflammatory cell infiltration. In the VD group, hepatocyte vacuoles were reduced, and the degree of fatty liver degeneration was partially reduced. Masson staining was used to observe the liver fibrosis in each group (**Figure 4B**), and the results showed that compared with the NC group, the HFD group showed an increase in blue collagen fiber infiltration, and the VD group could reduce the symptoms of liver fibrosis. Meanwhile, vitamin D supplementation reduced liver weight caused by HFD (**Figure 4C**). In addition, both ALT and AST were significantly higher in the HFD group (*P*<0.01), which was reversed in the VD group (*P*<0.05).

We evaluated the effect of 1,25(OH)2D3 on palmitic and oleic acid-induced HepG2 fatty liver cell models by *in vitro* experiments. The CCK8 assay found that cell viability reached its highest when the concentration of 1,25(OH)2D3 was 10–7 M (**Figure 4F**). Oil red O staining showed a large amount of red lipid deposition in the cells of the HF group compared with the NC group, which was alleviated by supplementation with 1,25(OH)2D3 (**Figure 4G**). Finally, RT-PCR results showed that the expression of *PPAR γ* mRNA, *FAS* mRNA, and *fatty acid-binding protein 4* (*FABP 4*) mRNA was increased in the HF group compared with NC (*P*<0.05), whereas the expression of *PPAR γ* mRNA was decreased after 1,25(OH)2D3 treatment (*P*<0.05) (**Figures 4H–J**).

### 3.5 The effect of vitamin D on the intestinal barrier

**Figure 5:**
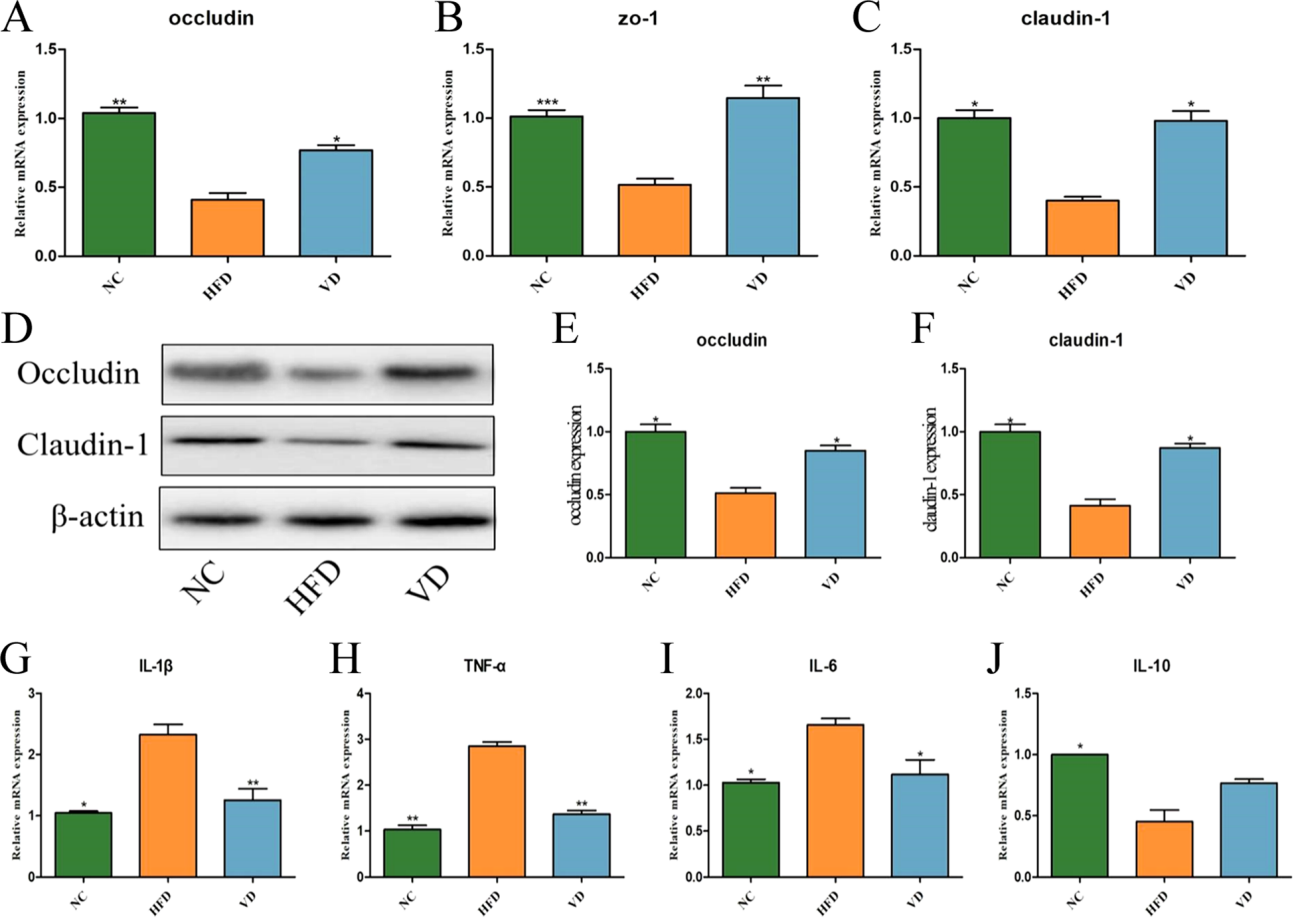
Intestinal barrier function and colonic tissue cytokine levels. Relative mRNA expression levels of *Occludin***(A)**, *ZO-1***(B)** and *Claudin-1***(C)**. **(D)** Western blot analysis of TJ protein expression in colon tissues. **(E)** Occludin protein quantification. **(F)** Claudin-1 protein quantification. Relative mRNA expression levels of *IL-1β***(G)**, *TNF-α***(H)**, *IL-6***(I)** and *IL-10***(J)**. Data are the mean ± SD (n=8). Compared with the HFD, *P<0.05, **P<0.01 and *** *P*<0.001.

High-fat diet-induced obesity is strongly associated with the integrity of the intestinal barrier (Brown et al., 2023). As shown in **Figures 5A–C**, RT-PCR results showed that *TJ* mRNA expression was significantly reduced in the HFD group compared to the NC group. Next, the expression of two tight junction (TJ) proteins, Occludin and Claudin-1, was reduced in the HFD group (**Figures 5D–F**). Meanwhile, vitamin D treatment reversed the HFD-induced reduction in the expression of these two proteins. On the contrary, vitamin D treatment reversed this one. The expression levels of cytokines in colonic tissues are shown in **Figures 5G–J**. The expression levels of *IL-1β*, *TNF-α* and *IL-6* mRNA were significantly higher in the HFD group compared with mice in the NC group (*P*<0.01). The VD group decreased the expression levels of *TNF-α*, *IL-1β* and *IL-6* mRNA, but had no effect on *IL-10* mRNA expression.

### 3.6 The effect of vitamin D on the gut microbiota

**Figure 6:**
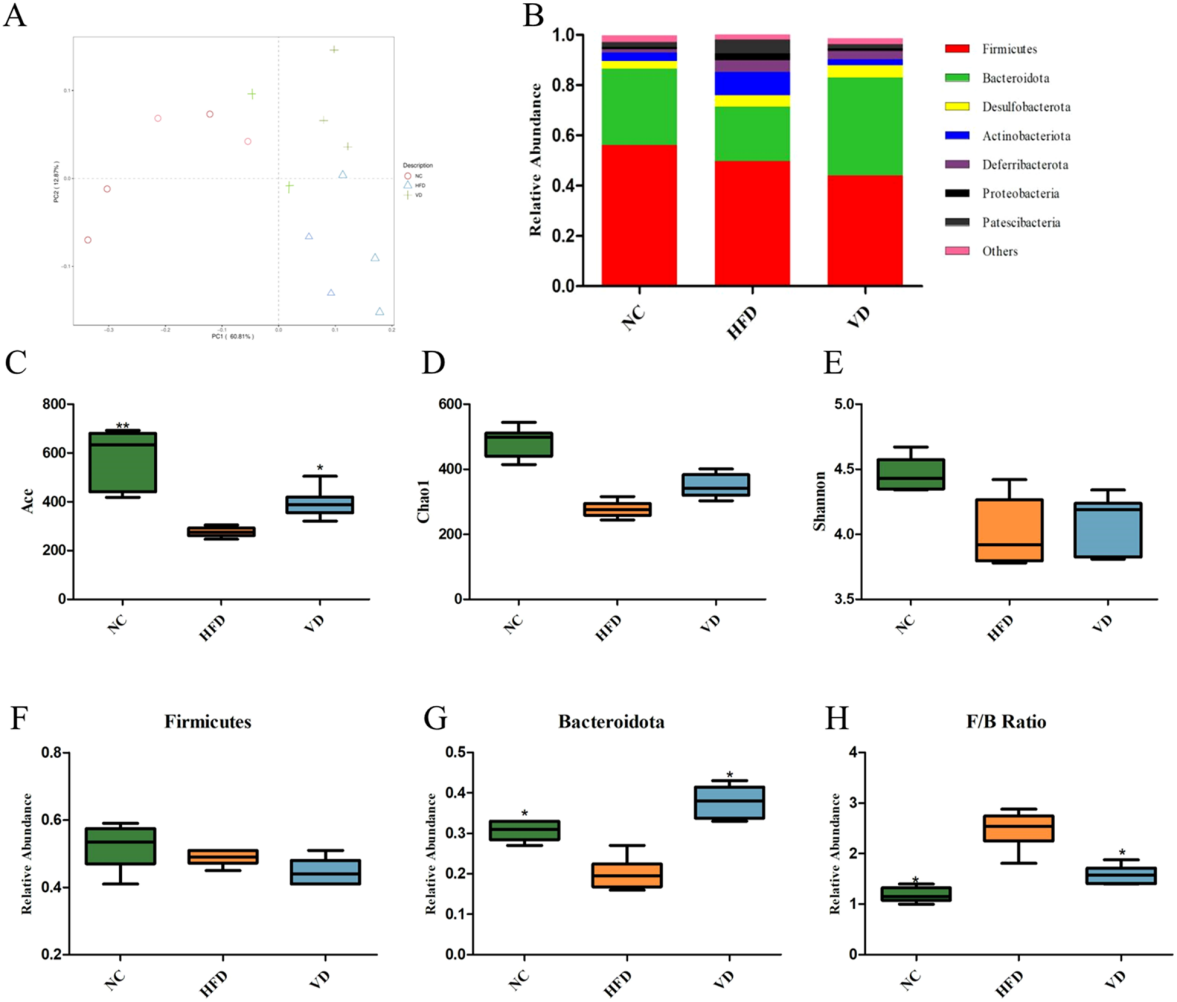
Gut microbiota analysis. **(A)** PCoA analysis. **(B)** Composition of gut microbiota at the phylum level. **(C)** Ace index. **(D)** Chao1 index. **(E)** Shannon index. **(F)** Firmicutes abundance. **(G)** Bacteroides abundance. **(H)** F/B ratio. Data are the mean ± SD. Compared with the HFD, *P<0.05 and **P<0.01.

Next, the gut microbiota of each group of mice was analyzed. Principal coordinate analysis (PCoA) based on unifrac showed differences among the three groups (**Figure 6A**), specifically, the distance between the VD group and the NC group was closer than that between the VD group and the HFD group. Comparing the gut microbiota composition at the phylum level (**Figure 6B**), it was found that in all three groups, Firmicutes, Bacteroidetes, and Actinobacteria accounted for over 75% of the total microbiota. Compared with the NC group, the proportion of Bacteroidetes was reduced in the HFD group (31.27% vs 21.32%). Notably, vitamin D treatment increased the proportion of Bacteroidetes (36.74% vs 21.32%), while the number of Firmicutes in the VD group also decreased (47.32% vs 43.67%). Compared with the NC group, the Ace index and Chao1 index were significantly reduced in the HFD group, while the Ace index increased in the VD group, and the Chao1 index showed a trend toward increase but without statistical significance (**Figures 6C–E**). An increase or decrease in the Firmicutes/Bacteroidetes (F/B) ratio of the gut microbiota is considered an ecological imbalance. We found that compared with the NC group, the Firmicutes proportion in the HFD group showed no significant difference, while the Bacteroidetes proportion decreased (**Figures 6F, G**). Vitamin D intervention could reduce the HFD-induced F/B ratio (*P*<0.05, **Figure 6H**).

**Figure 7:**
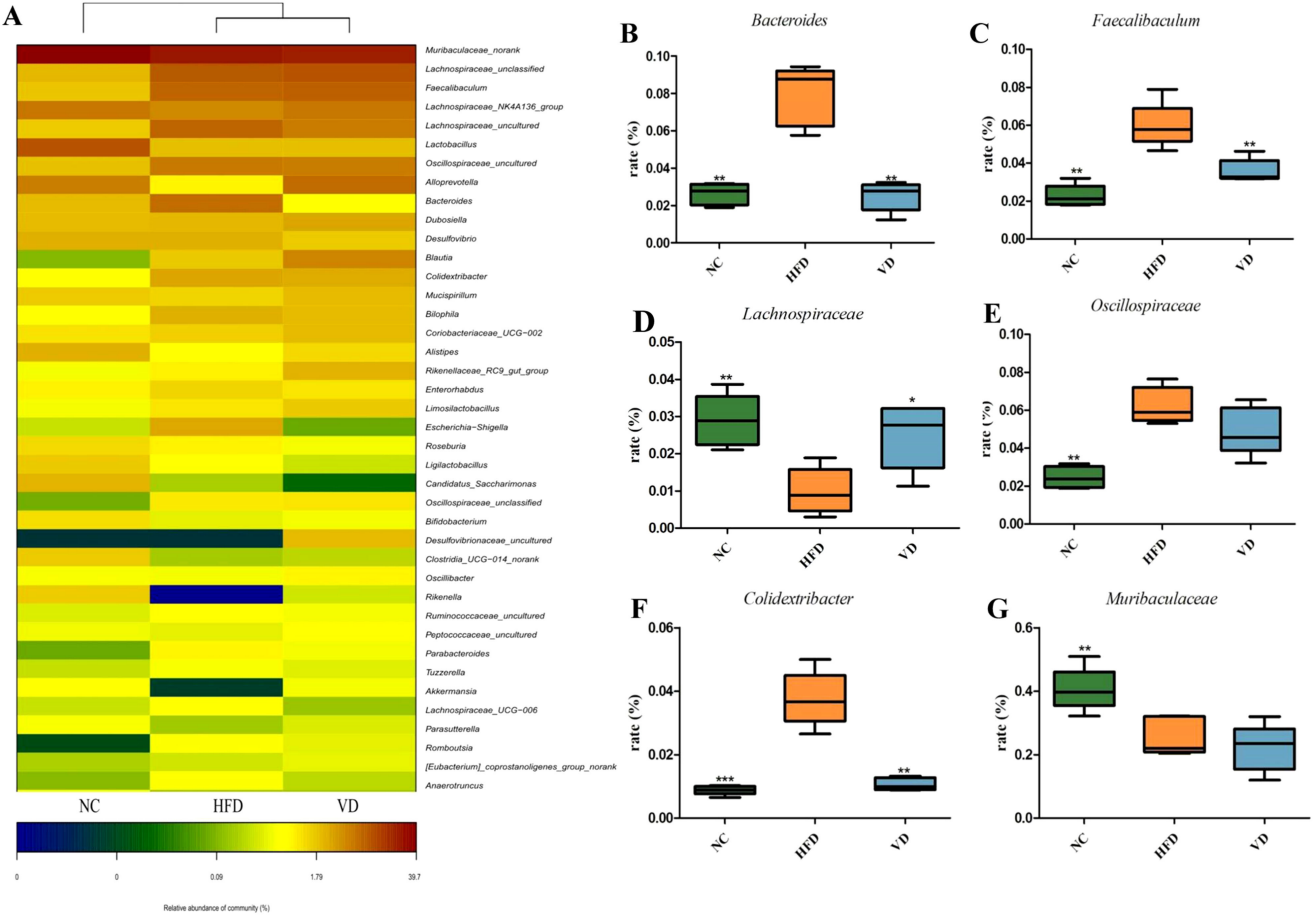
Microbial genus-level analysis. **(A)** Relative abundance of microbial genera. Relative proportions of Bacteroides **(B)**, Faecalibaculum **(C)**, Lachnospiraceae **(D)**, Oscillospiraceae **(E)**, Colidextribacter **(F)**, and Muribaculaceae **(G)**. Compared with the HFD, *P<0.05, **P<0.01 and *** *P*<0.001.

We then analyzed the relative abundance of the gut microbiota at the genus level, with a heatmap showing the abundance of the top 40 microbial groups (**Figure 7A**). Compared with the NC group, the HFD group showed increased relative abundance of *Faecalibaculum*, *Oscillospiraceae*, *Bilophila*, and *Coriobacteriaceae*, and decreased abundance of *Muribaculaceae*, *Lachnospiraceae*, *Bacteroides*, *Desulfovibrio*, and *Lactobacillus*. Vitamin D supplementation altered the abundance of *Lachnospiraceae*, *Bacteroides*, *Lactobacillus*, *Colidextribacter*, and *Blautia*. We selected six differentially expressed microbial taxa for further analysis. As shown in **Figures 7B–G**, compared with the NC group, the HFD group exhibited significantly increased abundance of *Bacteroides*, *Faecalibaculum*, *Oscillospiraceae*, and *Colidextribacter* (*P*<0.01). Vitamin D supplementation reduced the abundance of *Bacteroides*, *Faecalibaculum*, and *Colidextribacter*, but had no significant effect on the relative abundance of *Oscillospiraceae*. Vitamin D supplementation also increased the abundance of the beneficial gut bacterium *Lachnospiraceae* (*P*<0.05).

### 3.7 Correlation between gut microbiota and metabolic parameters

**Figure 8:**
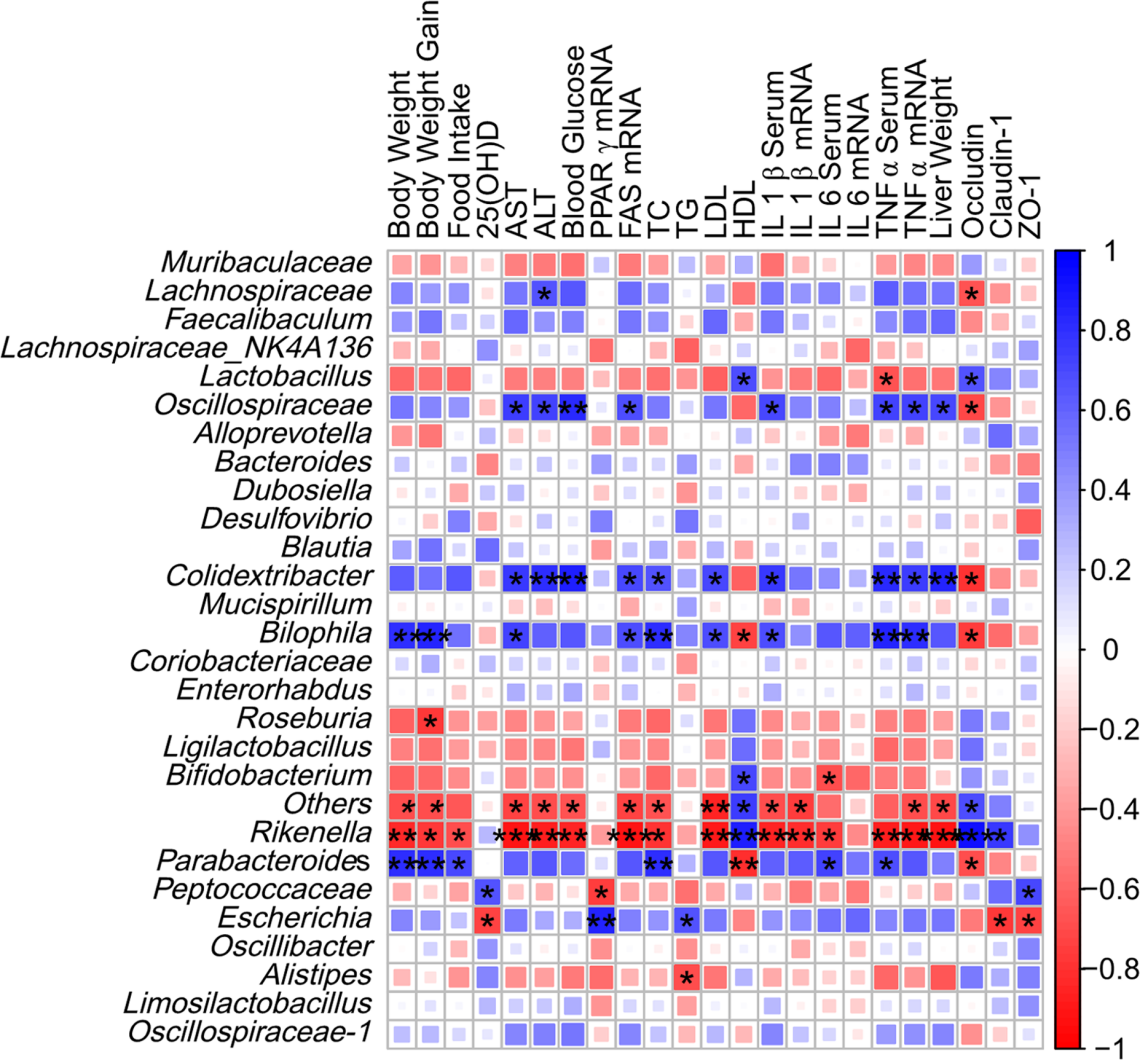
Spearman correlation analysis. Colors range from blue (positive correlation) to red (negative correlation). *P<0.05 and **P<0.01.

Spearman correlation analysis was used to explore the correlation between gut microbiota and metabolic parameters at the genus level (**Figure 8**). The results showed that *Lactobacillus* was positively correlated with HDL-c level and occludin protein expression, and negatively correlated with serum TNF-α level. *Oscillospiraceae* are associated with serum AST, ALT, and IL-1β levels. *Colidextribacter* was positively correlated with most of the lipid metabolism markers (TC, TG levels, and *PPAR γ* mRNA). Similarly, *Bilophila* and *Colidextribacter* had similar results. Notably, *Bifidobacterium* was positively correlated with serum HDL-c levels and negatively correlated with IL-1β levels. *Rikenella* was negatively correlated with most metabolic parameters, but *Parabacteroides* showed the opposite results. It is worth noting that the two bacterial species associated with serum 25(OH)D levels are *Pettococcaceae* and *Escherichia*. *Alistipes*, an important bacterial group regulating glucose metabolism, is associated with serum TG levels.

## 4 Discussion

Obesity and ageing are major health issues in today’s society. The prevention and management of obesity are particularly important for older adults. Older women have decreased estrogen levels, resulting in disorders of lipid metabolism and abnormal body fat distribution. During the reproductive period of women, the average level of total estrogen is 100–250 pg/ml. However, the concentration of estrogen in the postmenopausal cycle drops to 10 pg/ml. Due to estrogen deficiency and lipid metabolism disorders, older women have a higher risk of developing cardiovascular diseases (Hu et al., 2023). Dyslipidemia is very prevalent in older women, and its management is key to preventing increased cardiovascular morbidity and mortality worldwide. In recent years, there has been increasing interest in the non-skeletal effects of vitamin D, such as its potential prevention of diabetes, hyperlipidemia, and modulation of immune function (Giustina et al., 2024). Vitamin D deficiency is common in older women, which undoubtedly further exacerbates the risk of cardiovascular disease and dyslipidemia. The gut microbiota influences the development of obesity, and dysbiosis of the gut microbiota may be a major risk factor. Increasing evidence suggests that the gut microbiota may be a potential target for developing therapeutic agents for obesity and metabolic syndrome (Zhang et al., 2021; Artusa and White, 2025). The vitamin D/VDR signaling pathway plays an important role in the immune, genetic, environmental, and microbial aspects of human health and disease. In this study, we investigated the effects of vitamin D supplementation on lipid metabolism, inflammatory factors and intestinal microbiota in older female mice induced by high-fat diet. The results showed that vitamin D supplementation could improve the integrity of the intestinal barrier, regulate the imbalance of intestinal microbiota, further regulate abnormal blood lipid metabolism and cytokines, regulate the expression of fatty acid metabolism genes, and improve the degeneration of liver tissue and adipose tissue.

The decline in estrogen leads to abnormal fat distribution, characterized by the accumulation of visceral fat. Older women are at greater risk of obesity-related metabolic disorders than premenopausal women. Vitamin D insufficiency is more common in older women due to the rapid decline in estrogen protection of bones, reduced outdoor activity, and inadequate dietary intake (Kim, 2021). Chacko et al. conducted a cross-sectional analysis of 292 postmenopausal women aged 50–79 and found that serum 25(OH)D levels were associated with cardiovascular metabolic risk factors in postmenopausal women (Chacko et al., 2011). A randomized controlled trial found that supplementation with 4,000 IU of vitamin D reduced serum triglyceride levels in postmenopausal women with type 2 diabetes (Muñoz-Aguirre et al., 2015). Low vitamin D levels impair the nitric oxide signaling pathway in arteries, leading to dysfunction of vasodilators (Tare et al., 2011). In this study, we found that vitamin D supplementation reduced body weight, serum TC, TG levels, increased serum HLD-c levels, and improved liver steatosis and fibrosis symptoms in older female mice on a high-fat diet. Increased adipose tissue is associated with inflammation, and adipocyte hypertrophy may lead to increased synthesis and secretion of pro-inflammatory mediators such as TNF-α, IL-6, and IL-1β (Ding et al., 2012). The anti-inflammatory effects of vitamin D may be mediated through inhibition of the NF-κB signaling pathway, thereby reducing cytokine release and adipose tissue inflammation (Ding et al., 2013; Yerezhepov et al., 2024). As expected, our study found that vitamin D supplementation reduced serum IL-6 levels, and notably, the effect of vitamin D on reducing serum IL-1β and TNF-α levels was more pronounced. These indicators are highly correlated with key biomarkers of metabolic syndrome in elderly women (Razmjou et al., 2018; Schmitt et al., 2018), providing experimental evidence for the prevention of obesity-related lipid metabolism abnormalities through vitamin D supplementation in elderly women.

The accumulation of lipids in the livers of older female mice induced by a high-fat diet may be due to a lack of lipid utilization or adipokines involved in lipid consumption. Obesity is caused by the remodeling and proliferation of adipose tissue. Excessive fat deposition in obesity leads to an imbalance in the expression of adipokines in adipocytes. PPAR proteins mediate hepatic lipogenesis and hepatic steatosis, FAS is involved in fatty acid synthesis, and FABP 4 is involved in lipid transport, uptake, and metabolism (Zhang et al., 2022; Pratama et al., 2024; Barb et al., 2025). Vitamin D deficiency has been shown to be associated with increased PPAR expression and activity (Krisnamurti et al., 2023). Vitamin D influences fat formation by regulating the expression of CCAAT enhancer-binding protein α (C/EBP α) and PPAR γ through VDR-dependent mechanisms (Salehpour et al., 2021). C/EBP α and PPAR γ are two transcription factors associated with fat formation. PPAR γ mediates hepatic lipogenesis, and vitamin D receptors and PPAR are structurally similar, and while there may be a link between these two molecules, their link to hepatic steatosis is uncertain. Our study found that two genes involved in adipogenesis, *FAS* and *PPAR γ*, were more expressed in the liver of the high-fat diet group, and vitamin D supplementation affected the expression of *PPAR γ* mRNA in liver tissue. In addition, estrogen deficiency impairs PPAR α, and estrogen and vitamin D share similar functions and signaling pathways, which may suggest that the effects of estrogen and vitamin D deficiency on metabolic syndrome in older women may be consistent (Tidwell and Valliant, 2011).

The gut microbiota is essential for maintaining normal physiological functions in the mammalian gut, including regulating the immune system, eliminating toxic substances, and enhancing the intestinal barrier (Zhang et al., 2022; O'Riordan et al., 2025). Excessive fat intake (high-fat diet) can lead to ecological imbalance, intestinal barrier dysfunction, increased intestinal permeability, and leakage of toxic bacterial metabolites into the circulation. These changes in the microbiota lead to reduced butyrate production, impaired intestinal barrier integrity, and chronic inflammatory responses, which further inhibit lipid breakdown and promote adipocyte differentiation, resulting in excessive fat accumulation throughout the body. The gut has long been considered a major target for vitamin D (Guo et al., 2022). Vitamin D exerts its effects through VDR mediated mechanisms. On one hand, VDR binds to gut microbiota metabolites (such as short-chain fatty acids) to regulate the expression of tight junction proteins (occludin, claudin-1) in intestinal epithelial cells, thereby maintaining intestinal barrier integrity and modulating innate and adaptive immunity (Lin et al., 2023; Franco and McCoy, 2024). On the other hand, activated VDR inhibits the NF-κB signaling pathway, reducing the release of pro-inflammatory factors (IL-1β, TNF-α) while downregulating PPARγ expression, thereby improving lipid metabolism (Pham et al., 2021; Wallace et al., 2023). Additionally, vitamin D exerts adaptive immune suppression by inhibiting Th1/Th17 cells (Reich et al., 2014). When the vitamin D signaling pathway is disrupted, the development of inflammatory bowel disease demonstrates the importance of vitamin D/VDR in maintaining intestinal homeostasis (Kellermann et al., 2020; Li et al., 2023). These mechanisms collectively constitute the molecular pathway through which vitamin D regulates lipid metabolism via the gut microbiota. This study found that key bacterial communities regulated by vitamin D possess distinct metabolic functions. *Lachnospiraceae* and *Lactobacillus* produce short-chain fatty acids, promoting lipolysis and energy metabolism, while *Muribaculaceae* degrade dietary fiber and reduce intestinal inflammation (Bradley and Haran, 2024; Zhang et al., 2025). Vitamin D supplementation increased the abundance of beneficial gut microbiota, including *Lachnospiraceae*, *Lactobacillus*, *Colidextribacter*, *Oscillospiraceae*, and *Muribaculaceae*, while reducing the abundance of *Escherichia-Shigella*, *Colidextribacter*, *Bilophila*, and *Blautia*. Furthermore, vitamin D supplementation effectively reduced the abundance of gut microbiota associated with obesity and inflammation. In older female mice on a high-fat diet, vitamin D supplementation improved the stability of the intestinal epithelial barrier structure, increased the abundance of beneficial bacteria, reshaped the composition of the gut microbiota, and restored gut microbiota dysbiosis. This study is based solely on an older female mouse model. Due to differences between mice and humans in gut microbiota composition, hormone levels, and metabolic characteristics, the results can not be directly extrapolated to humans. We will subsequently conduct clinical studies targeting elderly women to verify the metabolic regulatory effects of vitamin D and to determine its clinical applicability.

## 5 Conclusions

In summary, vitamin D has beneficial effects on obesity and metabolic abnormalities in older female mice fed a high-fat diet. Vitamin D supplementation can regulate lipid levels and inflammatory factors in older female mice fed a high-fat diet, improve hepatic and adipose tissue degeneration, further regulate intestinal microbiota imbalance, and alleviate lipid metabolism abnormalities induced by a high-fat diet. The complex interactions between vitamin D, lipid metabolism abnormalities, and the gut microbiota are clearly understudied. Future research will primarily focus on conducting clinical trials of vitamin D interventions in elderly women, integrating metabolomics and fecal microbiota transplantation to thoroughly investigate the mechanisms underlying microbiota-host interactions.

## Data Availability

The datasets presented in this study can be found in online repositories. The names of the repository/repositories and accession number(s) can be found in the article/**Supplementary Material**.
