# Supplementary material for: Vitamin D alleviates obesity-related metabolic abnormalities by modulating the gut microbiota in older female mice on a high-fat diet
Source: Front Cell Infect Microbiol. 2025 Dec 10;15:1703497. doi: 10.3389/fcimb.2025.1703497 (PMC12727612; doi:10.3389/fcimb.2025.1703497)
Supplement: Supplementary file 1 [file Table1.docx]

**Table S1. Ingredient composition of the experimental diets**

| Ingredients | NC（g/100g） | HFD（g/100g） |
| --- | --- | --- |
| Casein | 20 | 20 |
| Corn starch | 39.7 | 25 |
| Sucrose | 10 | 20 |
| corn oil | - | 5 |
| lard | - | 20 |
| Mineral mixture | 3.5 | 3.5 |
| Vitamin C | 1 | 1 |
| cellulose mixture | 5 | 5 |
| Choline bitartrate | 0.3 | 0.3 |
| Methionine | - | 0.2 |
| L -Cystine | 0.3 | - |
| Dextrose | 13.2 | - |
| Soybean oil | 7 | - |
