# Supplementary material for: Vitamin D alleviates obesity-related metabolic abnormalities by modulating the gut microbiota in older female mice on a high-fat diet
Source: Front Cell Infect Microbiol. 2025 Dec 10;15:1703497. doi: 10.3389/fcimb.2025.1703497 (PMC12727612; doi:10.3389/fcimb.2025.1703497)
Supplement: Supplementary file 2 [file Table2.docx]

**Table S2. RT-PCR primer design**

| **Target gene** | **Primer sequence** |
| --- | --- |
| Fas | F: TCTGGTTCTTACGTCTGTTGC  R: CTGTGCAGTCCCTAGCTTTCC |
| Ppar γ | F: GGAAGACCACTCGCATTCCTT  R: GTAATCAGCAACCATTGGGTCA |
| Lpl | F: TTGCCCTAAGGACCCCTGAA  R: TTGAAGTGGCAGTTAGACACAG |
| Fabp4 | F: TTCGCCACCAGGAAAGT  R: ACGCCCAGTTTGAAGGA |
| TNF-α | F: TGGGACAGTGACCTGGACTGT  R: TTCGGAAAGCCCATTTGAGT |
| IL-1β | F: TTGACGGACCCCAAAAGATG  R: AGAAGGTGCTCATGTCCTCA |
| IL-6 | F: CTTCTTGGGACTGATGCTGGTGAC  R: AGGTCTGTTGGGAGTGGTATCCTC |
| IL-10 | F: GGTTGTCGTCTCATTCTGAAAGA  R: GGTAGAGGACCCAAGTTCGTTAAGA |
| Occludin | F: ATGTCCGGCCGATGCTCTC  R: TTTGGCTGCTCTTGGGTCTGTAT |
| ZO-1 | F: ACCCGAAACTGATGCTGTGGATAG  R: AAATGGCCGGGCAGAACTTGTGTA |
| Claudin-1 | F: AGATACAGTGCAAAGTCTTCGA  R: CAGGATGCCAATTACCATCAAG |
| β-actin | F: CCTCACTGTCCACCTTCCA  R: GGGTGTAAAACGCAGCTCA |

F: Forword; R: Reverse
